# Exploring the diverse career trajectories of general practice graduates in the French-speaking part of Belgium: An interview study

**DOI:** 10.1080/13814788.2021.1933938

**Published:** 2021-06-16

**Authors:** Anne-Laure Lenoir, Sophie Leconte, Marion Cayn, Frédéric Ketterer, Christiane Duchesnes, Béatrice Fraipont, Lou Richelle

**Affiliations:** a Department of General Practice, Université de Liège, University hospital of Sart-Tilman, Liège, Belgium; b Academic Centre of General Practice, Université catholique de Louvain, Louvain, Belgium; c Department of General Practice, Université Libre de Bruxelles, Erasme Campus, Brussels, Belgium

**Keywords:** General practice, career choice, career path, qualitative research

## Abstract

**Background:**

Several European countries face a shortage of general practitioners (GPs), in part due to GP attrition. Most studies of GP attrition have focussed on why GPs decide to leave. Yet understanding why GPs decide to remain may also elicit potential interventions to reduce attrition.

**Objectives:**

This study examined GP graduates’ career trajectories and underlying decisions to elucidate the factors influencing GP attrition.

**Methods:**

We conducted semi-structured interviews of early to mid-career general practice graduates having completed training in Belgian French-speaking universities between 1999 and 2013. We sampled participants from three categories: full-time GPs, part-time GPs, no longer working as GPs. We analysed each participant’s career trajectory and broke it down into major phases. We performed thematic analysis of the factors influencing participants’ trajectories. We compared and contrasted trajectories to develop a typology of career trajectories.

**Results:**

We identified six types of career trajectories: ‘stable’ (never considered leaving general practice), ‘reaffirmed’ (had considered leaving but made substantial changes whilst remaining), ‘reactional reorientations’ (had left to escape the challenges of general practice), ‘inspired reorientations’ (had left to pursue a different job), ‘reorientations out of loyalty’ (had never wanted to practice as GPs and had remained true to their original professional aspirations) and ‘mobiles’ (valued change and did not want to set-up practice).

**Conclusion:**

Reasons GPs leave the profession are multiple. The typology that emerged indicates that only some of the career trajectories would benefit from interventions to reduce attrition such as improving working conditions and providing psychological support for GPs.

KEY MESSAGES

- Dissatisfactory working conditions are a driver of attrition in general practice but evolving professional aspirations and GPs’ inability to alter working conditions also play a role.
- By distinguishing six career trajectories, we propose different targets for interventions to reduce attrition by increasing job satisfaction and helping GPs overcome common challenges.

## Introduction

Countries with a strong primary care system have better health outcomes and fewer health inequalities [1]. General practice is a major player in primary care [2,3]. Unfortunately, in some Organisation for Economic Co-operation and Development (OECD) countries, policy-makers have raised concerns about current or anticipated shortages in the general practice workforce [4]. While, in many European countries, the overall number of doctors increased between 2000 and 2017, the proportion of general practitioners (GPs) decreased by more than 10% in Belgium, Germany, Norway, Poland, Austria and the Czech Republic, and more than 20% in Denmark, Estonia, the United Kingdom and Ireland.

Shortages of general practitioners can stem from insufficient recruitment or excessive attrition. Efforts to prevent or reduce GP shortages have focussed primarily on recruitment, specifically on increasing the number of medical students choosing to specialise in general practice [5].

However, attrition, i.e. GPs leaving general practice before retirement, is also a major issue. For example, 21.5% of general practice graduates who had completed training in Belgian French-speaking universities between 1999 and 2013 were not practising as GPs in 2015 [6]. Similarly, in the United Kingdom, a survey conducted in 2017 in the South of England found 18% of GPs intended to leave general practice in the next two years [7].

Attrition in general practice has been mostly linked to low job satisfaction associated with issues of excessive workload, poor work-life balance, difficulties in work organisation, lack of support, disillusionment with the health system or negative media portrayals [5,7–11]. Yet, not all GPs who leave general practice are dissatisfied [12], suggesting that decisions to leave could bring into play other factors such as ill-health, family reasons or a desire to pursue another activity [7,10,13].

Most studies were survey studies. Career decisions are typically complex, dynamic phenomena that unfold over time [14]. Survey studies may not capture the interplay of multiple factors over time, and qualitative methods may be more appropriate. A systematic review of factors that affect GPs’ decisions to leave direct patient care found only six qualitative studies focussed on the reasons why GPs leave the profession [15]. Many of the findings from these studies also centred around job satisfaction, but they highlighted the contextual factors influencing job satisfaction such as professional relationships and workplace culture [15]. Another qualitative study – excluded from the systematic review due to its language (French) – used career trajectory analysis [16]. By examining the career trajectories of GPs, Bloy was able to identify long-standing factors, that were present before the end of medical school, such as whether or not general practice had been participants’ first choice of speciality. However, she conducted her study in France where postgraduate training in general practice is mainly hospital-based limiting the transferability of her findings to countries where postgraduate training is largely primary-care based.

Previous studies have focussed on the reasons for leaving general practice and, to our knowledge, no studies have investigated reasons for remaining. Although attrition and retention are two sides of the same coin, it seems plausible that understanding those who choose to remain in general practice may provide additional insights to support the design of interventions to reduce attrition. In the current study, we focus on the following questions: What are the early to mid-career trajectories of general practice graduates in the French-speaking part of Belgium? What are the decisions involved in shaping these trajectories? What factors do GP graduates identify as having influenced the decisions that shaped their career trajectories?

## Methods

### Design of the study

We conducted semi-structured interviews to explore the complexity of general practice graduates’ professional histories, focussing on their career choices, but situating them within their personal histories and contexts.

### Population and recruitment

We chose to focus on early to mid-career general practice graduates to examine factors that were likely still at play in the profession. In a previous study, we had targeted the same population by surveying the doctors who had graduated with an Advanced Master of General Practice degree from one of the Belgian French-speaking universities between 1999 and 2013 [6]. We had asked them for consent to be recontacted.

From those who had provided consent to being re-contacted, we selected participants from three categories based on their current professional activities: full-time GPs, part-time GPs, and no longer working as GPs (some of whom were still in clinical practice but in another speciality). Within these three groups, we also strove for diversity in terms of gender, year of graduation and, type of practice (solo, group practice with only GPs, or multidisciplinary group practice) for those who were still working as GPs. One of six members of the research team contacted potential participants by phone, explained the purpose of the study, and for those who were willing to take part, arranged a time and place for the interview.

Career trajectory analysis involves the development of a typology of career paths. Creating a typology requires a sufficient number of cases to identify clear patterns within the career trajectories described by participants. We anticipated more than one career type by group. Bloy interviewed 51 participants and found five career types. Based on these considerations, we initially determined that interviewing 20 participants in each of the three groups should provide sufficient information power [17]. Following analysis, we determined that we had reached data saturation and decided that we did not need to recruit more participants.

### Data collection

Semi-structured interviews were conducted between July and December 2016 by six members of the research team in a place chosen by the respondents. To limit the variability between interviewers, we held two meetings where we agreed upon common interview procedures and practised conducting interviews.

The interview guide explored the following four chronological phases: speciality choice, general practice training (residency), starting in general practice, ensuing career. It also included questions about the factors they felt had influenced their career decisions.

The interviews were on average forty-three minutes long and were recorded and transcribed verbatim by each interviewer.

### Data analysis

Three members of the team performed thematic analysis of the data between February and July 2017.

We used the chronological phases described above as an overarching structure for coding. Two members of the team performed inductive line-by-line coding independently for each transcript. They then categorised codes into broader themes. The first author then reviewed transcripts and the codebook and discussed these with the two coders. They discussed discrepancies and reached consensus on coding.

The first author identified the factors described by participants as influencing each career transition. Two team members then identified groups of participants who had similar career paths in terms of their decisions and timing of their decisions and who identified similar factors as having influenced their decisions. Thus, by combining the similarities in career paths and underlying factors, they created a typology of career trajectories.

### Ethics

This study was approved by the ethics committees in each of the three universities who took part (Université catholique de Louvain: 2014/517, Université Libre de Bruxelles: 2014/437, Université de Liège: B707201422436).

## Results

Of the 60 doctors who agreed to participate, 59 were interviewed and one cancelled the scheduled interview. Some had changed their activities between the time of the survey study and the interview. At the time of the interviews, 36 participants were still working as GPs (22 full-time, 14 part-time), and 23 had left general practice (eighteen were involved in other types of clinical practice, five had left clinical practice altogether). Table 1 summarises participant characteristics. At the time of the interviews, participants had graduated two to fourteen years ago.

**Table 1.** Participant characteristics (*n* = 59).

	*n*
Type of professional activity	
Full time in general practice	22
Part time in general practice (with or without other professional activities)	14
No longer working as GPs – practice in another speciality	18
No longer working as GPs – no clinical practice	5
Gender	
Female	38
Male	21
Number of years since graduation	
<5 years	15
5 to 10 years	21
>10 years	23
Type of general practice	
Group practice: multidisciplinary	12
Group practice: general practitioners only	14
Solo practice	10

**Figure 1.**
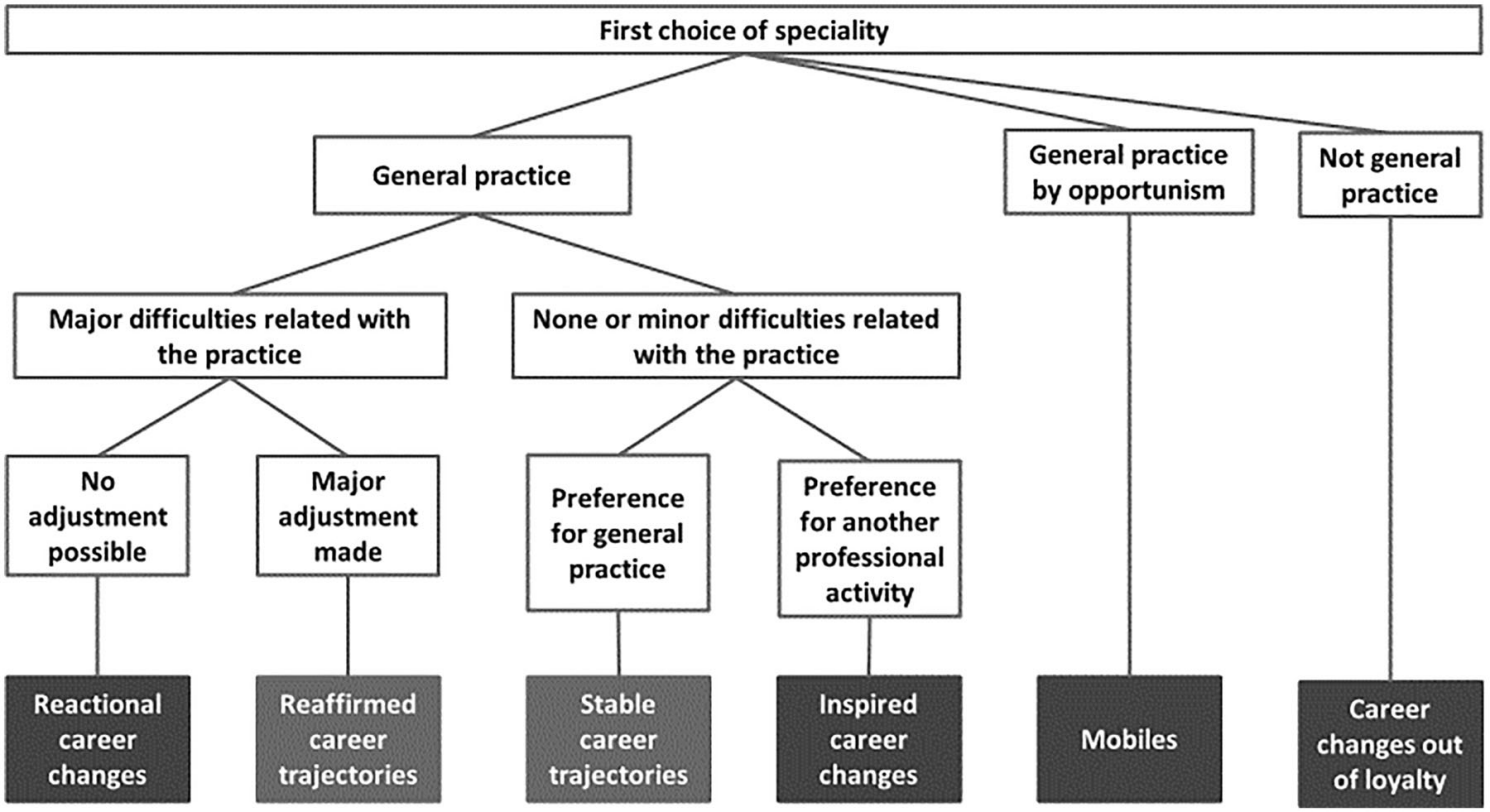
Graduates' trajectories.

We identified six types of career trajectories (Figure 1), which varied across the following dimensions: whether general practice was a first choice, whether graduates had begun practising as GPs following training completion, and whether graduates had ultimately remained in general practice. For those who switched to a different career, we further differentiated their trajectories based on the underlying reasons for the career change.

### Stable career trajectory

Participants with a ‘stable career trajectory’ had picked general practice as their first speciality choice and had never questioned this choice. They enjoyed the content of their jobs as GPs. Nonetheless, most of them found it challenging to balance their professional and private lives due to their heavy workload. They bemoaned the number of administrative tasks required of GPs, and felt that they lacked recognition for what they did. These challenges led them to adjust their working conditions over time by limiting their working hours, rejecting what they saw as unreasonable requests, and/or hiring administrative staff.

*‘Since I’ve really started working, I’m sure it’s what I wanted to do and what’s right for me. I love what I do even if, at times, I’m exhausted and it’s hard. But I have absolutely no regrets. General practice was really my choice, it’s really what I wanted to do, and I can’t see myself doing anything else, so, no, I’m super happy to be where I am now.’ (Female, full time, completed training less than five years previously)*

### Reaffirmed career trajectory

Participants with a ‘reaffirmed career trajectory’, had, at some point considered leaving general practice but in the end decided to remain in general practice. For some, this period of doubt had led them to take a temporary leave of absence. Their doubts stemmed from similar challenges as those encountered by participants with ‘stable’ career trajectories. However, on top of these difficulties, they had experienced an exacerbated sense of responsibility and a high emotional load in their work. They had made major changes to their type and/or location of practice, and/or their working hours, to mitigate these challenges. Some had diversified their professional activities, which they felt gave them a ‘break’ from general practice.

*‘After seven years [of medical school], it was too hard. The emotional impact of these illnesses [with a psychosocial component] was probably too much for me to handle. I was burnt out. And so, yeah, it played a role in my choice at some point to do something entirely different from medicine […] My changes, they might look like radical changes, but without these supervisions [with a therapist]; I don’t know how I would have managed. […] Taking stock now, I’m very happy that I made these difficult changes, on the one hand, and also I feel really fulfilled in my practice.’ (Male, full time, completed training five to ten years previously)*

### Reactional career change

Participants in the ‘reactional career change’ group had encountered the same difficulties as those with ‘reaffirmed career trajectories’ but these challenges had led them to change careers, sometimes as soon as they had completed their training. Most of these participants had suffered from burnout and had had to take a leave of absence. Most had left general practice after that but some had first attempted to alter their working conditions to no avail significantly. Participants in this group had found that their new career offered a more satisfying work environment, and none of them envisaged a return to general practice.

*‘I really had a burnout from general practice. Even talking about it… In fact, it’s very difficult, I think, for a woman to deal with everything that general practice requires. To manage the tasks, in terms of time, listening, everything and then family life. And I looked for something else while continuing general practice, but I could see it wasn’t working, I needed something else.’ (Female, left general practice, completed training five to ten years previously)*

### Inspired career change

Participants in the ‘inspired career change’ group, had become interested in a different career or activity. Their interest had either developed slowly over time or triggered by a specific event, leading to the discovery of this activity as a possible career option. Their career change had more to do with pursuing an alternative job than escaping general practice.

*‘I was struck by the case of a particular patient, because I noticed that he was a psychiatric patient, and, I would always run over time when I saw him. I really enjoyed it. I felt that something special was happening. […] So I thought, let’s go, I’ll go into psychiatry. It’s really a positive choice, psychiatry, I’m not someone who fled general practice because I didn’t like it.’ (Male, left general practice, completed training less than five years previously)*

### Reorientations out of loyalty

Participants in the fifth group had never intended to work as GPs. General practice had either been a fall-back plan for those who had not been selected in another speciality or a stepping stone to a field of practice, such as tropical medicine, sports medicine, or school health services. These fields require specific training but are not medical specialities per se, and general practice is one, if not the only, route to practising in these fields.

*‘I had set my mind on internal medicine, but my grades being what they were, I wasn’t selected. […] I always kept a foot in the hospital door. There are a whole other set of factors which meant that I liked the hospital environment. I was almost destined to work in a hospital. There is nothing that can be done to stop that. I’m an internist at heart, I couldn’t do anything else. […] In fact, when I finished my first year of hospital medicine there, they offered to hire me as a hospitalist GP, and, of course, I accepted.’ (Male, left general practice, completed training five to ten years previously)*

### Remaining mobile

Finally, participants in the sixth group sought to retain their mobility. From as early as the end of medical school, these ‘mobile’ participants had chosen to avoid settling down in a practice type or location. They had opted to specialise in general practice because of the shorter length of training and the perceived wealth of options open to them afterwards. Their career trajectory was not the result of careful planning but rather a series of opportunities.

*‘For me, if I have my own general practice, I’m putting down roots, it’s over. It’s like having a mortgage, a house and a family… I feel like I’m losing my mobility, my freedom, even if in fact it’s not necessarily the case. I know that a part of me would see it like that. I need to be able to move. If I put down roots, I feel like I’m going to suffocate from the inside.’ (Male, left general practice, completed training five to ten years previously)*

## Discussion

### Main findings

We interviewed general practice graduates, who had either remained or left general practice. Choosing to leave general practice was not always a response to the inherent challenges of general practice. We found that leaving general practice could also represent a pre-existing desire for professional mobility or the discovery of a new professional aspiration. In fact, for those who had never aspired to be general practitioners, leaving general practice was consistent with their initial professional ambitions.

Conversely, choosing to remain in general practice was not the result of complete job satisfaction. Many of those who were still working as GPs described similar causes of dissatisfaction than those who had left and had at some point questioned their choice of career. In other words, the same causes – challenging working conditions – did not lead to the same effects for all general practice graduates. Some simply made minor adjustments to their working conditions, while others made major changes, and others still transitioned to different careers. Our findings suggest that participants differed in the emotional toll they perceived general practice to take and in their ability to sufficiently alter their working conditions.

### Interpretation of the results in relation to existing literature

Our results extend those of previous studies on attrition in general practice. Like other studies, we found that some GPs may leave general practice because of its inherent challenges but others may leave because of a desire to pursue a different job [5,7,9,10,15,16], whether it’s one that they had always wanted to pursue or one they had just discovered an interest in [7,10,16]. Career changes are facilitated by the versatility of the degree in general practice, which provides access to a broad range of professional activities [16].

Our findings are largely consistent with Bloy’s in terms of the career trajectories of graduates who subsequently left general practice. She identified five career trajectories: stable careers that remained consistent with initial choice (whether general practice or not), shift to pursue a new professional interest, to switch to a more suitable job, switch to escape general practice, and careers that remain unstable. However, we did not identify substantive differences between those who had chosen to shift to pursue a new professional interest versus to start a more suitable job. Unlike Bloy, we also examined the career trajectories of those who remained in general practice and found that while some had never really questioned their career choice, others had, and had only remained because they had been able to sufficiently alter their working conditions. Thus, both those who reaffirmed their decision to work in general practice and those who rejected general practice, encountered significant challenges, albeit to varying degrees. While these groups may appear at opposites in terms of attrition versus retention, their paths were similar and led to a specific juncture where personal and contextual factors led to different outcomes.

A systematic review found that four job-related factors play a major role in decision-making about rejecting general practice: workload, job (dis)satisfaction, work-related stress and work-life balance [18]. They also highlight the importance of support and adaptation, which is consistent with our results.

### Implications for policy and research

Our findings point to various interventions to target these different reasons for leaving general practice. The first set of interventions could target job satisfaction. Our findings suggest the following as potential targets: decreasing the administrative burden, improving work-life balance, and increasing recognition from patients, colleagues from other specialities, and the State. However, working conditions should not be the only target of interventions because, as our findings highlight, the relationship between working conditions, job satisfaction, and attrition is not linear.

Another set of interventions should seek to help GPs to overcome common challenges, through increased organisational and psychological support throughout their career. Many participants reported having suffered from professional exhaustion. Despite the limitations of self-reported data, it seems reasonable to assume that interventions known to reduce burnout in doctors, either through psychological means (stress management, small group discussion) or organisational means (e.g. reduced working hours) could have a positive impact on retention in general practice [19]. Physicians tend to neglect their health and reluctant to seek help [20,21]. The medical culture, to which trainees are exposed from the beginning of medical school, contributes to these behaviours [22,23]. Our findings certainly point to challenges occurring during postgraduate training and the transition to independent practice, suggesting that interventions to prevent burnout should be implemented during training and in practice.

Finally, our findings suggest that not all cases of attrition are preventable: some graduates never intended to practice as GPs and are likely to find ways to fulfil their professional aspirations outside general practice. While some career paths suggested that some individuals may be more prone to leaving general practice, i.e. those who did not pick general practice as their first choice, those who want to remain mobile, we do not advocate for interventions to exclude them in selection processes. Given the shortage of GPs, it would be unfortunate to exclude those who could, despite being in these groups, nonetheless end up remaining in general practice. Our study highlights how these decisions evolve and may change. Our previous survey study on the professional activities of recently-graduated GPs found that those who had not picked GP as their first choice represented 9.4% of all of those still in practice [6].

Some of our proposed targets for interventions to reduce attrition have evidence regarding their effectiveness. A systematic review found that interventions targeting job satisfaction such as increasing autonomy, strengthening support and recognition, decreasing working hours and workload, were effective in reducing attrition, whereas retainer schemes, improving training capacity (i.e. subspecialisation and portfolio careers), financial incentives to remain in practice, and new ways of working (i.e. part time, reduced availability, and job mobility) were not [5]. The relationship between actual working conditions and job satisfaction is not straightforward; it is mediated by individuals’ perceptions and reactions to these conditions that matter. In our study, many GPs described the same sources of initial dissatisfaction but managed them differently, leading to different career decisions.

Future studies should examine the effectiveness of the additional interventions suggested by our findings, such as providing support to GPs throughout their careers to help them identify the sources of satisfaction/dissatisfaction in their job and find individualised ways to increase job satisfaction and prevent professional burnout.

### Strengths and limitations of the study

We purposefully used semi-structured interviews to understand the meaning that individuals gave to their own choices and experiences. Although we aimed to produce a typology, we examined career trajectories as a process and explored the factors underpinning career decisions.

However, interviews provide an individual’s reconstruction from memory. Memory is prone to biases. Individuals are more likely to remember facts or events with a high emotional charge or that involved more cognitive processing [24]. Moreover, narrated storylines involve selecting key plot features that individuals deem relevant and/or socially acceptable [24]. Storylines can also constitute a kind of *post hoc* rationalisation that provides meaning and consistency to individuals’ sense of their past and present [24].

We interviewed many participants, which enabled us to identify clusters of career trajectories from a diverse dataset. However, we do not claim that we have identified all possible types of career trajectories. For example, our sampling strategy focussed on the extent to which GPs were still practising but did not specifically recruit based on speciality preference. None of our participants who had remained in practice was from the group for whom general practice was not their first choice.

Furthermore, we interviewed early and mid-career participants. Their careers were by no means over and further transitions may occur in the future. Future studies could prospectively follow cohorts of general practice graduates and collect data longitudinally to provide ‘real time’ data on career trajectories as they unfold.

## Conclusion

Our findings highlight the multiple reasons GPs leave the profession. Some are amenable to interventions such as reducing the administrative burden on and working hours of GPs, and providing psychological support for GPs. Further research should collect data prospectively to minimise recall bias; and further disentangle the complex interacting factors that influence career trajectories.

## Supplementary Material

Interview Guide

COREQ Checklist
