# Supplementary material for: Exploring the diverse career trajectories of general practice graduates in the French-speaking part of Belgium: An interview study
Source: Eur J Gen Pract. 2021 Jun 16;27(1):111–8. doi: 10.1080/13814788.2021.1933938 (PMC8211135; doi:10.1080/13814788.2021.1933938)
Supplement: Interview Guide [file IGEN_A_1933938_SM6633.docx]

**Interview Guide**

1. Could you tell me about your academic background? Why did you choose to study medicine?
2. What made you choose a general practice residency?
   1. What attracted you?
   2. What were you afraid of?
   3. Were there particular people or events that influenced your choice?
3. Could you tell me about your residency?
   1. What did you particularly enjoy?
   2. What kinds of difficulties did you face?
   3. Did it meet your expectations? How was it different?
4. Then, could we talk about your career after graduation in general practice?

If they didn’t practice general practice after graduation:

a. Why didn’t you practise general practice?

b. What did you do after your residency?

c. Were you already thinking about a reorientation during your studies?

d. What made you decide?

e. Was it a difficult decision to make? What convinced you to do it?

If they practised general practice:

- 1. Did the beginning of your practice correspond to what you had imagined? If not, how was it different?
  2. What did you like about the job?
  3. What was more difficult to live with?

If they continued to practise general practice:

1. Does your current practice look like your early practice?
2. If not, what changes have taken place? How have you adapted your practice or personal organisation? Why? Were there particular events that disturbed you or, on the contrary, reinforced your choices?

If they have left general practice:

1. When did you stop practising general practice?
2. Why? Reasons, anteriority of the questioning or the will to stop, particular event, temporality, decision (difficulty, choice, evidence?)
3. What aspects of the profession do you regret? And those you are relieved to no longer have to face it?
4. And what about your current professional situation, does it suit you or are you thinking of changing it?
5. Do you have any other comments?

Thank you
