# Supplementary material for: Exploring the diverse career trajectories of general practice graduates in the French-speaking part of Belgium: An interview study
Source: Eur J Gen Pract. 2021 Jun 16;27(1):111–8. doi: 10.1080/13814788.2021.1933938 (PMC8211135; doi:10.1080/13814788.2021.1933938)
Supplement: COREQ Checklist [file IGEN_A_1933938_SM6632.docx]

| **COREQ checklist** | | | |
| --- | --- | --- | --- |
| No | Item | Description | Manuscript |
| **Domain 1: Research team and reflexivity** | | | |
| ***Personal characteristics*** | | | |
| 1 | Interviewer/facilitator | Six members of the research team conducted semi-structured interviews in a place chosen by the respondents: ALL, MC, FK, CD, BF and LR. | Methods, data collection, §1 |
| 2 | Credentials | ALL: MD, PhD  MC: MD  FK: sociologist, PhD  CD: MD, PhD  BF: MD  LR: MD  SL: MD, PhD |  |
| 3 | Occupation | ALL: GP and lecturer (Department of General Medicine University of Liège)  MC: GP and researcher (Department of General Medicine Free University of Brussels)  FK: researcher (Department of General Medicine University of Liège)  CD: researcher (Department of General Medicine University of Liège)  BF: researcher (Academic Centre of General Medicine, Catholic University of Louvain)  LR: GP and researcher (Department of General Medicine Free University of Brussels)  SL: researcher (Academic Centre of General Medicine, Catholic University of Louvain) |  |
| 4 | Gender | ALL: female  MC: female  FK: male  CD: female  BF: female  LR: female  SL: female |  |
| 5 | Experience and training | ALL: experienced researcher  MC: junior researcher trained in qualitative research  FK: experienced researcher  CD: experienced researcher  BF: experienced researcher  LR: junior researcher trained in qualitative research  SL: experienced researcher |  |
| ***Relationships with participants*** | | | |
| 6 | Relationship established | Participants took part in a survey about the professional activities of the general medical graduates from all three French-speaking Belgian universities between 1999 and 2013 and was carried out beforehand by a team of researchers. | Methods, population and recruitment, §1, 2 |
| 7 | Participant knowledge of the interviewer | The information known about interviewers was their university affiliation and their occupation. |  |
| 8 | Interviewer characteristics | ALL: PhD on the topic of attraction and retention of general practitioners  The other researchers didn’t have any personal interest in this research. |  |
| **Domain2: study design** | | | |
| ***Theorical framework*** | | | |
| 9 | Methodological orientation and theory | We performed thematic analysis of the data. We used the chronological phases described above as an overarching structure for coding. We created a graphical representation of each participants’ career trajectory, using the chronological phases described above. We charted the factors described by participants as influencing each career transition. | Method, data analysis |
| ***Participant selection*** | | | |
| 10 | Sampling | Participants took part in a survey about the professional activities of the general medical graduates from all three French-speaking Belgian universities between 1999 and 2013 and was carried out beforehand by a team of researchers.  From those who had provided consent to being re-contacted, we selected participants from three categories based on their current professional activities: full-time GPs, part-time GPs, and no longer working as GPs (some of whom were still in clinical practice but in another speciality). Within these three groups, we also strove for diversity in terms of gender, year of graduation and, type of practice (solo, group practice with only GPs, or multidisciplinary group practice) for those still working as GPs. | Methods, population and recruitment, §1, 2 |
| 11 | Method of approach | Participants who had provided consent to being re-contacted in the previous study were contacted by phone to confirm their participation and make an appointment for the interviews. | Methods, population and recruitment, §1, 2 |
| 12 | Sample size | Of the 60 doctors who had agreed to take part, 59 were interviewed and 1 cancelled the scheduled interview. | Results, §1 |
| 13 | Non-participation | The interviews were carried out voluntarily following up on the previous phase; it is impossible to know whether the graduates who chose to participate in the study were different from those who declined to take part (381 out of the 1,240 graduates who answered the census).  Only one participant cancelled the scheduled interview. | Methods, population and recruitment, §1, 2 |
| ***Setting*** | | | |
| 14 | Setting of data collection | Six members of the research team conducted semi-structured interviews in a place chosen by the respondents | Methods, data collection, §1 |
| 15 | Presence of non-participants | Anyone was present during the interviews. |  |
| 16 | Description of sample | We selected participants from three categories based on their current professional activities: full-time GPs, part-time GPs, and no longer working as GPs (some of whom were still in clinical practice but in another speciality). Within these three groups, we also strove for diversity in terms of gender, year of graduation and, type of practice (solo, group practice with only GPs, or multidisciplinary group practice) for those still working as GPs. | Methods, population and recruitment, §1 |
| ***Data collection*** | | | |
| 17 | Interview guide | The interview guide explored the following 4 chronological phases: speciality choice, general practice training (residency), starting in general practice, ensuing career. It also included questions about the factors they felt had influenced their career decisions. | Methods, data collection, §2 |
| 18 | Repeat interviews | There weren’t repeat interviews |  |
| 19 | Audio/visual recording | The interviews were recorded and transcribed verbatim by each interviewer. | Methods, data collection, §3 |
| 20 | Fields notes | There weren’t field notes. |  |
| 21 | Duration | The interviews were on average forty-three minutes long. | Methods, data collection, §3 |
| 22 | Data saturation | We first recruited 20 participants in each of the three groups. Given our aim to create a taxonomy of career trajectories and in line with Bloy’s study, we estimated that this number should provide sufficient information power (14). Following analysis, we determined that we had reached data saturation and decided that we did not need to recruit more participants. | Methods, population and recruitment, §3 |
| 23 | Transcripts returned | Transcripts didn’t return to participants for comment or correction. |  |
| **Domain 3: Analysis and findings** | | | |
| ***Data analysis*** | | | |
| 24 | Number of data coders | Three members of the team performed thematic analysis of the data. | Method, data analysis, §1 |
| 25 | Description of coding tree | We used the chronological phases described above as an overarching structure for coding. Two members of the team performed inductive line-by-line coding independently for each transcript. They then categorised codes in broader themes. The first author then reviewed transcripts and the codebook and discussed these with the two coders. They discussed discrepancies and reached consensus on coding.  The first author then created a graphical representation of each participants’ career trajectory, using the chronological phases described above. She charted the factors described by participants as influencing each career transition. Two team members then compared and contrasted the charts to identify and characterise groups of similar career trajectories, and create a typology. | Method, data analysis, §2,3 |
| 26 | Derivation of themes | Cf. 25 |  |
| 27 | Software | The thematic analysis was managed by Excel. |  |
| 28 | Participant checking | Participants didn’t provide feedback on the finding. |  |
| ***Reporting*** | | | |
| 29 | Quotations presented | Quotations were presented in the results. Each of them was identified. |  |
| 30 | Data and findings consistent | Presented data and findings were consistent. |  |
| 31 | Clarity of major themes | The first author then created a graphical representation of each participants’ career trajectory, using the chronological phases described above. She charted the factors described by participants as influencing each career transition. Two members of the team then compared and contrasted the charts to identify and characterise groups of similar career trajectories, and create a typology | Method, data analysis, §3 |
| 32 | Clarity of minor themes | This research aimed to identify the main career path typologies. For this reason, all career path had to match with one of the typologies. |  |
